# Medium for the Production of *Bacillus*-Based Biocontrol Agent Effective against Aflatoxigenic *Aspergillus flavus*: Dual Approach for Modelling and Optimization

**DOI:** 10.3390/microorganisms10061165

**Published:** 2022-06-06

**Authors:** Vanja Vlajkov, Stefan Anđelić, Ivana Pajčin, Mila Grahovac, Dragana Budakov, Aleksandar Jokić, Jovana Grahovac

**Affiliations:** 1 Faculty of Technology Novi Sad, University of Novi Sad, 21000 Novi Sad, Serbia; ivana.pajcin@uns.ac.rs (I.P.); jokic@uns.ac.rs (A.J.); 2 Faculty of Technical Sciences, University of Novi Sad, 21000 Novi Sad, Serbia; stefan.andjelic@uns.ac.rs; 3 Faculty of Agriculture, University of Novi Sad, 21000 Novi Sad, Serbia; mila.grahovac@polj.edu.rs (M.G.); dragana.budakov@polj.edu.rs (D.B.)

**Keywords:** bioprocess, ANN, RSM, modelling, optimization, biocontrol

## Abstract

One of the leading limiting factors for wider industrial production and commercialization of microbial biopesticides refers to the high costs of cultivation media. The selection of alternative sources of macronutrients crucial for the growth and metabolic activity of the producing microorganism is a necessary phase of the bioprocess development. Gaining a better understanding of the influence of the medium composition on the biotechnological production of biocontrol agents is enabled through bioprocess modelling and optimization. In the present study, after the selection of optimal carbon and nitrogen sources, two modelling approaches were applied to mathematically describe the behavior of the examined bioprocess—the production of biocontrol agents effective against aflatoxigenic *Aspergillus flavus* strains. The modelling was performed using four independent variables: cellulose, urea, ammonium sulfate and dipotassium phosphate, and the selected response was the inhibition-zone diameter. After the comparison of the results generated by the Response Surface Methodology (RSM) and the Artificial Neural Network (ANN) approach, the first model was chosen for the further optimization step due to the better fit of the experimental results. As the final investigation step, the optimal cultivation medium composition was defined (g/L): cellulose 5.0, ammonium sulfate 3.77, dipotassium phosphate 0.3, magnesium sulfate heptahydrate 0.3.

## 1. Introduction

The presence of aflatoxigenic strains in maize fields represents a global food-safety issue and a topic of great economic and public-health interest [1,2]. Since they are widely spread in nature, affecting numerous crops of industrial importance, the contamination control strategy is an urgent question to be addressed [3,4]. Biocontrol agents are recognized as a good balance between effectiveness in pest management, and on the other hand, securing environmental stability and sustainable food production [5,6]. 

The global biopesticide market evolution is supported by strong ecological and political initiatives [7]. On the other hand, the high production costs still represent the bottleneck in reaching the full commercialization capacity. The contribution of media preparation costs to the overall costs structure of biotechnological production refers to around 70% [8]. The design of a viable bioprocess solution scalable to the industrial level production strongly depends on the efficiently performed optimization step in terms of medium composition [8,9,10]. Defining alternative sources of macronutrients and their optimal concentrations is considered as an inevitable phase of microbial biopesticides production development [11]. The starting point of designing technology suitable for scale-up is finding an adequate producing microorganism [12,13]. The multiple selection criteria for the most potent biocontrol agent rely on the phytopathogen suppression effect but also on other characteristics important from the point of view of creating a viable bioprocess solution [14,15]. Members of the *Bacillus* genus are an excellent example of producing microorganisms considered suitable candidates for industrial-level biotechnological production. As one of the most important factors is the ability of the *Bacillus* spp. to utilize nutrients from the alternative inexpensive substrates [16,17,18].

The optimization step in the technology development is preceded by the bioprocess simulation considered as model-based representation of the examined system. The Response Surface Methodology (RSM) is a commonly and successfully applied modelling tool used in production processes of a wide range of biotechnological products, including antibiotics, enzymes, biopolymers, and biofuels [19,20,21]. The Artificial Neural Networks (ANN) approach is the method of choice widely used in bioprocess modelling due to the characteristic possibility of adapting to different examined systems. The key difference in the ANN modelling approach compared to the conventional methods is described by flexibility and a lack of restrictions on the relationship type between dependent variables and various input parameters [22]. Based on the available dataset, ANN transforms the independent variables into the predicted responses with the possibility of adjusting network factors [23]. 

The first aim of the study was to determine the optimal combination of carbon and nitrogen sources for biosynthesis medium preparation. Considering the previously mentioned importance of lowering the overall costs of the production, the selection of the potential carbon and nitrogen sources was completed by choosing those that represent common components of different industrial waste streams, to gain an insight into the possibility of their further usage as a medium basis. The following investigation step included modelling of nutrients’ concentration influence on the antimicrobial activity based on two methods, RSM and ANN, and finally, optimization of the quantitative content of the medium for the production of biocontrol agents.

## 2. Materials and Methods

### 2.1. Producing Microorganism and Inoculum Preparation

The producing microorganism, *Bacillus* sp. BioSolAfla, used for cultivation-medium optimization was previously isolated from the rhizosphere of *Phaseolus vulgaris* and selected among 76 *Bacillus* strains as the most efficient biocontrol agent in *Aspergillus flavus* suppression [3]. The 16S rRNA sequencing and VITEK2 Compact System identification indicated the highest similarity of the producing microorganism to the members of operational group *Bacillus amyloliquefaciens*. The inoculum preparation included transferring the loopful bacterial biomass to Erlenmayer flasks containing nutrient broth (50 mL) (HiMedia, Mumbai, India), and cultivation on a rotary shaker at 28 °C, 170 rpm, under spontaneous aeration for 24 h. The same experimental conditions were applied in the second step of inoculum preparation, when the liquid culture was transferred to the Erlenmayer flasks of higher volume containing the same commercial medium (150 mL) and cultivated for another 24 h. Inoculation of cultivation media for biosynthesis (50 mL) was performed by adding 10% (*v*/*v*) of the prepared inoculum. 

### 2.2. Selection of Carbon and Nitrogen Sources—Media Composition and Cultivation Conditions

The cultivation medium in the phase of optimal carbon source selection included (g/L): carbon source (10.0), yeast extract (3.0), (NH_4_)_2_SO_4_ (3.0), K_2_HPO_4_ (1.0), MgSO_4_·7H_2_O (0.3) and pH value was adjusted to 7.0 ± 0.2 prior to sterilization performed by autoclaving at 121 °C and 2.1 bar (20 min). Varied carbon sources included glycerol, starch, maltose, glucose, lactose, cellulose, and fructose. The cultivation media in the next phase of optimal nitrogen source selection contained cellulose as a carbon source, while varied organic nitrogen sources were yeast extract, peptone, tryptone, urea, L-glutamic acid, and all the other components remained the same as it was in the previous investigation step. In both investigation steps, cultivation was carried out on a rotary shaker for 96 h, at 28 °C, with an agitation rate of 170 rpm (KS 4000i control, IKA^®^ Werke, Staufen, Germany).

### 2.3. Experimental Design, Modelling (RSM and ANN) and Optimization

The media contents for the cultivation of the selected producing microorganism were defined according to the Box–Behnken experimental design (Table A1, Appendix A) by varying the initial concentration of four factors at three levels (g/L): cellulose (5–35), urea (0–5), ammonium sulfate (0–5), and dipotassium phosphate (0.5–4.5). The cultivation media also included magnesium sulfate heptahydrate added in the same concentration in all 27 combinations, 0.3 g/L): The effects of the selected independent variables were examined by following the values of the dependent variable (response)—the inhibition-zone diameter—as the direct indicator of biocontrol agent activity. 

The initial step before the statistical modelling included the analysis of the obtained experimental data, to determine possible inconsistencies, eliminate noise, and define an adequate modelling approach. The modelling was performed by applying the Response Surface Methodology (RSM) and Artificial Neural Networks (ANN) approach. 

In the first modelling method, experimental data were fitted using the second-degree polynomial function according to the equation:

*Y* = *b*_0_ + *b*_1_ · *X*_1_ + *b*_2_ · *X*_2_ + *b*_3_ · *X*_3_ + *b*_4_ · *X*_4_ + *b*_12_ · *X*_1_ · *X*_2_ + *b*_13_ · *X*_1_ · *X*_3_ + *b*_14_ · *X*_1_ · *X*_4_ + *b*_23_ · *X*_2_ · *X*_3_ +*b*_24_ · *X*_2_ · *X*_4_ + *b*_34_ · *X*_3_ · *X*_4_ + *b*_11_ · *X*_1_^2^ + *b*_22_ · *X*_2_^2^ + *b*_33_ · *X*_3_^2^ + *b*_44_ · *X*_4_^2^ (1)

where *Y* is the predicted response—the inhibition-zone diameter—*b*_0_ is the intercept coefficient, *b*_i_ is a linear coefficient, *b*_ii_ is a quadratic coefficient, and *b*_ij_ is interaction coefficient, while *X*_i_ are previously described independent variables—initial concentrations of cellulose, urea, ammonium sulfate, and dipotassium phosphate. 

The statistical significance of the regression model coefficients was estimated according to the generated *p*-values. Factorial ANOVA (analysis of variance) was used for estimation of the experimental data fitting by analysing the R^2^ (coefficient of determination) and statistical significance of the generated models based on *p*-values and F-values All statistical analysis were performed at the statistical significance level of 0.05, using Statistica 13.5 software (Tibco Software Inc., Carslbad, CA, USA). 

Prior to the ANN modelling step, the experimental data were subjected to data augmentation. All data have been normalized to the range [0, 1] based on predetermined experimental limitations of the medium components’ concentrations, as well as possible values of the response surface in the instruments (Table 1). The normalization brings a model extrapolative limitation but also significantly reduces the possibility of a model bias towards higher values of attributes as well as the problem of exploding and vanishing gradients during the optimization. The optimal architecture of the autoencoder network used for augmentation was obtained by searching the parameter space for the number of neurons in the output layer of the encoder, embedding layer, selecting the activation function in the hidden layers of the encoder and decoder [elu (Exponential Linear Unit), relu (Rectified Linear Unit), sigmoid], learning rate [0.01–0.03] and learning-rate degradation speed [0.5–0.95]. The number of epochs was set to a maximum of 50,000 iterations, with a premature stop in case of extremely small error variation. The chosen architecture for modelling included a multilayer perceptron model for encoder and decoder, with a four-neuron embedding layer. The modelling was performed using ScikitLearn 0.24.2 (https://scikit-learn.org/stable/ (accessed on 20 February 2022)) and Tensorflow 2.5.0 (https://www.tensorflow.org/ (accessed on 20 February 2022)) software in Python 3.8.

Training of the data augmentation network was performed using the entire dataset, bringing the same data to the input and output of the auto-encoder, for both isolates separately. An auto-encoder network involves two connected networks including an encoder and a decoder. The process was initiated by compressing the original data into a short code ignoring the background signals using the encoder. After that, the decoder decompresses the given code to generate data as close as possible to the input data. The trained autoencoder can be used to generate new data following the statistical trends of the original data set by bringing random values to the input of the trained decoder. The values of the output from the encoder layer, the input to the decoder layer, which are simulated, do not have to be found in any form in the original set. The use of network autoencoders is also a robust way to eliminate noise that can be found in the data.

Once the model was trained, the decoder network was separated from the architecture, and using randomly sampled numbers from the range [−1, 2.5], corresponding to the slightly expanded range seen on the embedding layer in the original data set, a new extended dataset has been generated. Data at the output of the decoder have the statistical characteristics of the original dataset and enable the generation of an arbitrary number of new data samples. For the purpose of training further predictors, 10,000 new samples were generated for each isolate using neural networks. An experiment was defined to find the optimal architecture of this model, similar to the one from the previous step.

A multilayer perceptron with three hidden layers was used as the architecture of the neural network of the predictive model. To optimize the model for both isolates, a fivefold cross-validation [5,6,7,8,9,10] was performed while changing activation functions in hidden layers [elu, relu, sigmoid], the learning rate [0.01–0.04], and degradation rates [0.3–0.95]. The original set of experimental values was used as a final validation set, which exists only in implicit form in the training data. In this way, the validation of the predictive model has also validated the success of data augmentation. The original set of experimental values was used as a final validation set. This set exists only in an implicit form in the training data, since the training data were generated from the autoencoder and do not contain the exact data points from the original dataset. In this way, the validation of the predictive model has also validated the success of data augmentation, as the metrics would confirm that the original dataset can be fully reconstructed using the predictor that was trained without them.

The optimization step of cultivation medium content was performed employing Desirability Function methodology in the Design-Expert 8.1. (Stat-Ease, Inc., Minneapolis, MN, USA).

### 2.4. Antimicrobial Activity Assay

The suspension of test microorganisms (phytopathogenic strains *Aspergillus flavus* SA2B and *Aspergillus flavus* PA2D) was prepared by adding the fungal biomass in sterile saline to achieve the final spore concentration of 10^5^ CFU/mL. Sabourad maltose agar media (Himedia Laboratories, Mumbai, India) were melted and tempered (50 ± 1 °C) and, before pouring into the Petri dishes, inoculated using 1 mL of the previously prepared fungal suspensions. The well diffusion method in triplicate tests was employed to evaluate the antimicrobial activity of the cultivation broth samples (100 µL) obtained after 96 h of cultivation of the producing microorganism, *Bacillus* sp. BioSol021, against the phytopathogenic strains. The incubation was performed at 30 °C for 96 h and followed by the inhibition-zone diameter measurement.

## 3. Results

### 3.1. Selection of the Optimal Carbon and Organic Nitrogen Sources

The obtained results of the inhibition-zone diameters were subjected to the one-factor analysis of variance at a significance level of 95%. The results of the analysis indicated a statistically significant influence of the selected carbon sources on the values of the inhibition-zone diameters (*p*-values less than 0.05) (Table 2). Homogenous groups of carbon sources and statistical significance of differences among the obtained inhibition-zone diameters were established using Duncan’s multiple range test and the results are shown in Table 3. The same statistical analysis was performed for the results obtained in the phase of organic nitrogen source selection, which has also indicated the significant effect of the selected organic nitrogen sources in the cultivation medium on the obtained values of inhibition-zone diameters. The results of the ANOVA are shown in Table 4 and Duncan’s multiple range test results are given in Table 5.

### 3.2. The Explorative Analysis of the Experimental Data

**Figure 1:**
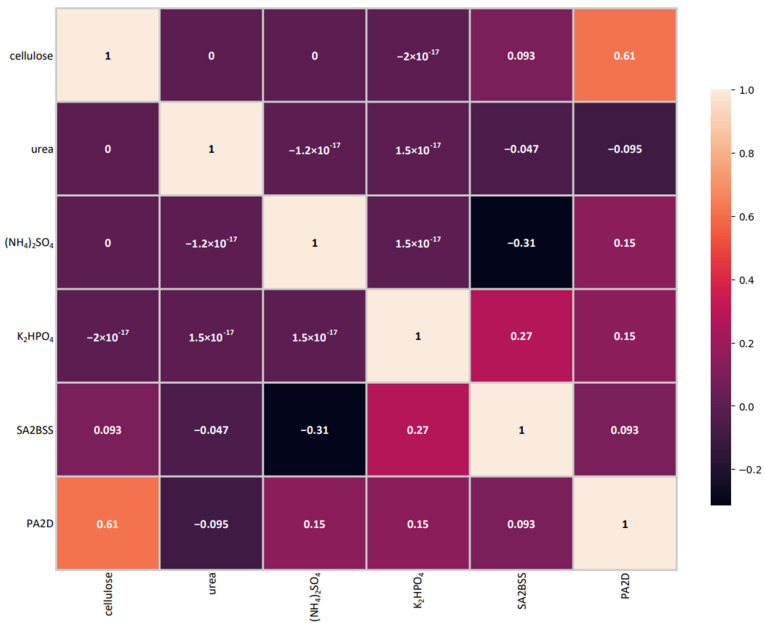
Correlation matrix of the experimental values used in the medium composition modeling.

The correlation analysis results of all attributes of the dataset are given in Figure 1. 

### 3.3. RSM Model

The effect of the cultivation medium components (cellulose as a carbon source, urea as an organic nitrogen source, (NH_4_)_2_SO_4_ as an inorganic nitrogen source, and K_2_HPO_4_ as a phosphorus source) on the antagonistic activity of the producing strain was evaluated. The second-degree polynomial equations were used to fit the experimental data. The obtained linear, quadratic and interaction regression coefficients and their *p*-values for both models including the inhibition-zone diameters against the aflatoxigenic strains *Aspergillus flavus* SA2BSS and *Aspergillus flavus* PA2DSS as the dependent variables are presented in Table 6 and Table 7.

Analysis of variance (ANOVA) was performed for both models to determine their statistical significance with the confidence level of 95% (Table 8 and Table 9). For a better understanding of the effects of the nutrient content on the inhibition-zone diameters, the response surface plots were generated as a graphical illustration of the relationship between the variables. The visual presentations of the selected responses were generated when two independent variables were varied and the other two remained constant with the values corresponding to the central point of the Box–Behnken experimental plan (Appendix B and Appendix C).

### 3.4. Augmentation Model

The optimal model architecture, which resulted from a parameter space search, consists of two multi-layer perceptrons for the encoder and the decoder, with an embedding layer of four neurons. The performance metrics of the selected model were determined by a mean square error (MSE) and a coefficient of determination. The optimal training hyper-parameter values were 0.03 for the innitial learning rate and 0.8 for the learning degradation rate, and an elu has been selected as the optimal activation function in every layer except the last one, where a sigmoid function has been used. Table 10 shows MSE and R^2^ values of the data augmentation auto-encoder.

### 3.5. ANN Model

The performance metrics of the selected model were determined by the mean square error and the coefficient of determination (Table 11). The optimal values of the training parameters, determined in the previous step, were 0.03 for the training speed with a degradation rate of 0.8, and elu activation function in all layers except the output, where the sigmoid function was used. Data regularization was performed by the dropout with a probability of 5% as well as l2 regularization with a coefficient of 0.01. The use of a stronger regularization has improved the ability of the model to generalize and reduced the possibility of overfitting, which is a significant risk, especially in the case of a small data sample. The training results of the best models, as well as their hyperparameters are shown in Table 11. The performance of the models was calculated over the validation set.

The coefficient of determination and the mean square error over the training set were 0.98 and 10^−5^, respectively, and the high accuracy over the validation set confirmed that the model fits the original data well. A slight deviation from the original set indicates a possible improvement in the model generalization. 

The visually presented responses in case of ANN model predictions were obtained when two independent variables were varied and the other two remained constant with the values corresponding to the central point of the Box–Behnken experimental plan (Appendix D and Appendix E).

### 3.6. RSM and ANN Models Comparison

The adjusted coefficients of determination for the models of tested aflatoxigenic strains, *Aspergillus flavus* SA2BSS and *Aspergillus flavus* PA2DSS, generated using two modelling approaches, RSM and ANN, are given in the Table 12.

### 3.7. Optimization

The Desirability Function methodology was employed to optimize the initial content of carbon, organic and inorganic nitrogen, and phosphorous sources in the medium used for the cultivation of Bacillus sp. BioSolAfla. The considered optimization set and the obtained results are shown in Table 13. All the independent and dependent variables were assigned the same weighting factor 3 (range 1–5).

## 4. Discussion

The commercial media, commonly used in the initial investigation steps in bioprocess development for the production of biocontrol agents, are considered an unsuitable choice when it comes to the stages of production scale-up. The explanation lies in the unaffordable price, limiting the possibility of shifting to industrial-scale production and product commercialization. It proves the necessity to make additional efforts to find alternative sources of nutrients providing favorable conditions for the growth and metabolic activity of selected isolates. A step further in the formulation of complex media based on natural components is the identification of waste streams rich in appropriate nutrients for microbial growth and metabolic activity [8,24,25,26,27,28]. 

It is important to point out that the nutritional requirements of microorganisms are closely related to a particular strain. The ability to assimilate certain nutrients in a qualitative and quantitative manner is one of the main determinants of the strains’ nature and their metabolic activity. In the present study, the chosen carbon sources were selected as representatives of the common waste streams which could be possibly evaluated in the further investigation as components of the cultivation medium. The initial optimization step involved the standard OFAT approach (one factor at a time), considering the variation of one factor. In the first case, it was the carbon source, and after that, organic carbon source, while the other components of the cultivation medium were unchanged [19]. The selection of a carbon source as a key macronutrient for the growth and reproduction of the producing microorganism and its metabolic activity is considered the primary step in cultivation-medium optimization. The assimilation capacity of certain carbon sources, as well as their nature and origin, affect the biomass growth, and type and yield of metabolic products. The influence happens in both directions, including repression and maximization of the production intensity [19]. 

Speaking generally, members of the genus *Bacillus* are characterized by the ability to utilize different carbohydrates as the main sources of carbon and energy as well [29]. As was previously mentioned, from an economic point of view, the additional criterion for the selection of potential carbon sources considered their availability and presence in the waste streams of various branches of industry. Glucose is a commonly preferred carbon source of most bacterial species [29]. Investigation into the potential of glucose for use as a carbon source is almost inevitable in research aiming at medium optimization [30,31,32,33]. Glucose is also a common component in food-industry waste streams with an emphasis on wine, fruit, and vegetable processing. These waste streams are also a significant source of fructose, which is in addition to glucose, the most often included in the optimization of the medium composition for the cultivation of *Bacillus* species [34]. Members of the *Bacillus* genus are also credited with a well-known ability of enzymatic activity and utilization of nutrients from different substrates [18,35]; starch, maltose, and lactose were also considered as potential carbon sources. Cellulose is the most abundant carbohydrate in nature and, at the same time, the most important renewable resource. It is of economic interest to find a solution regarding the valorization of significant amounts of cellulose as a component of agrocellulose waste into the products with added market value, such as microbiological biopesticides [36,37,38,39,40]. Maltose is an important component of waste streams generated in beer production [41], while lactose is present in waste streams of the dairy industry. Maltase and lactase are also important groups of extracellular hydrolytic enzymes produced by isolates of the *Bacillus* genus [42,43] that enable carbon utilization originating from these substrates. In a similar way, waste streams of flour and bakery products are rich in starch, which can also be included as a potential carbon source, taking into account the amylolytic activity of the *Bacillus* genus representatives [44]. Crude glycerol is a common by-product of the biodiesel industry. The growing trend of biodiesel production implies the necessity of creating a solution for the generated waste to be converted into valuable products [45,46]. In this study, the statistical significance evaluation of the influence of carbon sources on antimicrobial activity was performed by employing the one-factor analysis of variance of the obtained results for inhibition-zone diameters, at a significance level of 95%. The results of the analysis indicated the statistically significant influence of selected carbon sources on the antimicrobial activity, and Duncan’s multiple range test was employed to define the homogenous groups, pointing out the best choices for the production of antimicrobial agents effective against aflatoxigenic *Aspergillus flavus* isolates. Cellulose and fructose were identified as carbon sources providing the highest antagonistic activity. The same statistical analysis was repeated in the case of organic nitrogen selection. The results indicated a statistically significant influence of nitrogen sources on the antimicrobial activity, and Duncan’s multiple range test showed that the optimal source would be urea. By performing a comparison of the pathogen inhibition effect, it was concluded that the optimal combination includes cellulose and urea as the main sources of macronutrients in the cultivation medium for biocontrol strain *Bacillus* sp. BioSolAfla.

The modelling of the cultivation medium for the production of biocontrol agents effective against aflatoxigenic *Aspergillus flavus* strains was performed by two approaches: RSM and ANN. The first modelling method considered defining the values of the intercept, linear, quadratic, and interaction coefficients which determine the influence of each independent variable on the defined response. The generated mathematical models in the form of the second-degree equations have described the behavior of the selected dependent variable depending on the number of independent variables. The main limitation of RSM is described by the possibility of assuming only quadratic correlations [23]. On the other hand, the ANN approach allows modelling of the experimental data using more complex correlations, while the model itself and its inner parameters are observed as a black box. Since the ANN method is based on training by experience, applying a new input to the network enables the generation of the new result according to the previous experience [23]. From the perspective of biotechnological production in general, the importance of RSM lies in the ability to define influence of certain factors and their interactions on the selected response and better understanding of complex relations within the observed system. On the other hand, the main limitation of the RSM is explained by the lack of possibility for extrapolation of the generated relationships for the factors out of the defined variable’s value range. 

The ANN approach overcomes the aforementioned limitations by generating the models that provide the data based on the deep statistical relations applicable to the generalized values. Applying this kind of modeling method is of particular importance when it comes to the investigation of the possibility of waste-stream valorization as alternative components of the cultivation medium, which could potentially contain nutrients in concentrations exceeding the range defined by the RSM model. A double modeling approach allows the achievement of deeper insight into the observed system, necessary for a better understanding of the influence of key parameters and their manipulation in further investigation steps, including optimization and bioprocess scale-up.

The experimental data used to perform the modeling were generated according to the Box–Behnken experimental design. Each independent variable was placed at one of three equally distanced value levels. The main advantage of using the reduced experimental design reflects in the significant resources savings while generating the necessary data to obtain a statistically valid mathematical model. The repeatability under the same experimental conditions was secured by performing the central point experiment in triplicate. 

Prior to modeling, the analysis of the obtained experimental values was performed to determine possible inconsistencies, eliminate noise, and define a suitable modelling approach. The first step of the analysis included determination of the correlation of all attributes of the dataset. The obtained results indicated a negligible correlation of the medium components’ concentrations, with the exception of the relationship between the response for the *Aspergillus flavus* PA2DSS isolate and cellulose. The correlation coefficient implied a medium statistical dependence and indicated the carbon source as a potentially moderately important factor. The correlation analysis has not indicated the need to ignore values from the dataset, and all measured attributes were used in modeling. 

The adequacy of the generated mathematical models obtained by RSM was per-formed by statistical significance analysis based on *p*-values and F-values. The low *p*-values and high F-values in the case of both models indicated their satisfactory fitting of the experimental data. The coefficients of the models for the responses regarding the antimicrobial activity against the both aflatoxigenic isolates, and their corresponding *p*-values, as well as the graphically illustrated response surfaces, indicated important insights regarding the significance of the selected nutrients and their interactions on the antimicrobial activity of the producing strain. 

The data-augmentation model, based on autoencoders, has displayed good performance and has shown to be invaluable for the downstream task of training the predictor model. The predictor model validation on the initial data displays a good capability for generalization and great performance when applied to unseen data. While the R^2^ value of the model was not higher than other methods, it is still implied that the data itself is inconsistent and the model learned correctly while not overfitting.

By making a comparison of the models generated using two different approaches based on their graphical illustrations, it could be concluded that in most cases they showed the similar influence of the selected factors on the observed response. The antagonistic effect of the producing microorganism is attributed to the dualistic mode of action, including the competitive activity of the biomass and antibiosis. It explains the nutrient requirements necessary for the bacterial cells’ growth, and on the other hand, production of secondary metabolites characterized by the antimicrobial activity against the selected phytopathogens. Besides the carbon source, the presence of nitrogen is necessary as it is a key macronutrient for both functions. On the one hand, there is a need to secure enough nitrogen for generating a high concentration of biomass, and on the other hand, for the synthesis of compounds that exhibit antimicrobial activity and belong to the group of peptides [19,47,48]. The obtained results also pointed out the importance of defining the adequate balance of the organic and inorganic nitrogen sources, as well as carbon and nitrogen sources in the medium [49]. The availability of phosphorus in the cultivation medium defines the duration of the exponential growth phase. The limitation of the phosphorus content results in the initiation of the stationary growth phase when the secondary metabolites are produced, indicating high antimicrobial activity. On the other hand, a higher phosphorus concentration favors biomass generation, contributing to the competitive activity of biocontrol agents [50]. 

By making a comparison of the results for models generated using the RSM and the methodology of ANN, where the values of the adjusted determination coefficients were 0.91 (RSM) and 0.83 (ANN) for the tested isolate *Aspergillus flavus* SA2BSS, and 0.89 (RSM) and 0.81 (ANN) for *Aspergillus flavus* PA2DSS (Table 12), it was concluded that, in this particular case, the second-order polynomial model better fits the experimental data. According to the obtained results the model generated by the RSM was used in the further phase of bioprocess optimization. The obtained RSM model better describes the existing experimental data with mathematical relations that allow a deeper understanding of the influence of defined variables and their mutual interactions on the observed responses. The negative aspect of the response surface methodology application refers to the limited experimental space and the variation of variables in an exclusively given range with the impossibility of extrapolation. The analysis of the observed system is completed by generating a model using the methodology of ANN that provides information on the influence of factors beyond the value range given by the experimental plan [51,52].

The following step in bioprocess solution development included optimization of the cultivation medium content. The Desirability Function methodology is one of the common optimization approaches involving the usage of a series of nonlinear algorithms to find the optimal solution defined by the optimization set goals [53]. The first step included determining the desired optimization goals related to the target value range of the observed responses. In the present study, the optimal solution for the cultivation medium content was defined by optimization set implying the maximization of the inhibition-zone diameters for both phytopathogenic strains, *Aspergillus flavus* SA2BSS and *Aspergillus flavus* PA2DSS, and minimizing the initial concentration of carbon and organic nitrogen sources. The maximization of the inhibition-zone diameter is related to the maximum efficiency of the biological control agent in suppressive activity against the studied phytopathogens. On the other hand, minimizing the concentrations of the basic components of the cultivation medium contributes to lowering the production costs and economic viability of the entire bioprocess. Based on the obtained results shown in Table 13, the goals of the optimization set were achieved by defining the following composition of the cultivation medium (g/L): cellulose, urea 0, ammonium sulfate 3.77, potassium hydrogen phosphate 0.3, magnesium sulfate heptahydrate 0.3. The obtained value of the total desired function was 0.75. The results of the cultivation medium optimization confirmed the previously established relationship between the independent variables and their impact on the selected responses based on the results obtained by applying the RSM. The results of the optimization set predicted the following responses’ values: inhibition-zone diameter for the strain *Aspergillus flavus* SA2BSS 30.66 mm, and 27.84 mm for the strain *Aspergillus flavus* PA2DSS.

The precondition for the commercialization of biological control agents is the definition of an economically acceptable medium enabling the production of a sufficient amount of the highly efficient final product at the industrial scale. The constituents of the medium have to meet the requirements regarding the nutritional needs of the production isolate for biomass growth, energy supply and metabolic activity. Representatives of the genus *Bacillus* have proven to be suitable candidates for scaling the production process to an industrial level, taking into account the ability to utilize nutrients from alternative and economically suitable substrates [54]. One of the examples of specific significance to the present study is proven cellulase activity [40,55,56] of *Bacillus* spp. This provides a promising basis for considering the usage of different sources of lignocellulosic material as possible components of cultivation medium suitable for biocontrol-agent production in an economically efficient way [57,58,59,60]. The results of the study demonstrate the promising basis for creating a viable bioprocess solution using a cultivation medium including an alternative carbon source and even without an organic nitrogen source, which is of great importance considering its high market prize.

## Figures and Tables

**Table 1:** Normalization ranges of attributes in ANN modelling. All of these ranges were scaled to the range [0, 1].

Attribute	Normalization Range Values
cellulose	0–50 (g/L)
urea	0–10 (g/L)
(NH_4_)_2_SO_4_	0–10 (g/L)
K_2_HPO_4_	0–10 (g/L)
*Aspergillus flavus* SA2BSS	10–40 (mm)
*Aspergillus flavus* PA2DSS	10–40 (mm)

**Table 2:** One-way ANOVA of inhibition-zone diameters obtained by testing antagonistic activity of the cultivation broth samples against the aflatoxigenic isolates in the carbon source selection investigation step.

Effect	SS	DF	MS	F-Value	*p*-Value
Inhibition-zone diameter (mm)	25,063.71	1	25,063.71	20,243.77	0.000000
Carbon source	70.95	6	11.83	9.55	0.000003
Error	43.33	35	1.24		

SS—sum of squares; DF—degree of freedom; MS—mean square.

**Table 3:** Mean values of inhibition-zone diameters obtained by testing cultivation broth samples against aflatoxigenic isolates in the carbon source selection investigation step.

Carbon Source	Inhibition-Zone Diameter (mm)
Starch	22.33333 ^a^
Lactose	23.83333 ^b^
Glucose	23.83333 ^b^
Maltose	24.16667 ^b^
Glycerol	24.33333 ^b^
Fructose	26.16667 ^c^
Cellulose	26.33333 ^c^

Values marked with the same letter are at the same level of statistical significance.

**Table 4:** One-way ANOVA of inhibition-zone diameter obtained by testing antagonistic activity of cultivation broth samples against the aflatoxigenic isolates in the nitrogen source selection investigation step.

Effect	SS	DF	MS	F-Value	*p*-Value
Inhibition-zone diameter (mm)	23,520.00	1	23,520.00	8204.651	0.000000
Nitrogen source	214.33	4	53.58	18.692	0.000000
Error	71.67	25	2.87		

SS—sum of squares; DF—degree of freedom; MS—mean squares.

**Table 5:** Mean values of inhibition-zone diameters obtained by testing the cultivation broth samples against the aflatoxigenic isolates in the nitrogen source selection investigation step.

Nitrogen Source	Inhibition-Zone Diameter (mm)
Peptone	25.00000 ^a^
Yeast extract	25.00000 ^a^
Tryptone	28.16667 ^b^
L-glutamic acid	30.33333 ^c^
Urea	31.50000 ^c^

Values marked with the same letter are at the same level of statistical significance.

**Table 6:** Coefficients of regression equation and their *p*-values for the diameter of the inhibition zone for *Aspergillus flavus* SA2BSS as test microorganism.

Coefficient	Value	*p*-Value
b_0_	38.98960	0.000000
b_1_	−0.33876	0.000287
b_2_	−1.49165	0.001542
b_3_	−1.59720	0.000931
b_4_	−3.70852	0.000007
b_12_	0.00000	0.999989
b_13_	0.00667	0.464301
b_14_	0.05278	0.000442
b_23_	0.25333	0.000443
b_24_	−0.01667	0.805301
b_34_	0.15000	0.042610
b_11_	0.00512	0.001688
b_22_	0.17111	0.002853
b_33_	0.03111	0.510096
b_44_	0.52779	0.000009

1—cellulose; 2—urea; 3—(NH_4_)_2_SO_4_; 4—K_2_HPO_4_.

**Table 7:** Coefficients of regression equation and their *p*-values for the diameter of the inhibition zone for *Aspergillus flavus* PA2DSS as test microorganism.

Coefficient	Value	*p*-Value
b_0_	24.21942	0.000000
b_1_	−0.10015	0.134429
b_2_	0.48611	0.179911
b_3_	1.52500	0.000769
b_4_	0.23958	0.609578
b_12_	−0.02000	0.031472
b_13_	0.00222	0.791376
b_14_	0.02500	0.031472
b_23_	−0.12000	0.031472
b_24_	0.05000	0.432879
b_34_	−0.25000	0.001589
b_11_	0.00414	0.004481
b_22_	0.00222	0.959338
b_33_	−0.10444	0.030777
b_44_	−0.01736	0.799053

1—cellulose; 2—urea; 3—(NH_4_)_2_SO_4_; 4—K_2_HPO_4_.

**Table 8:** ANOVA for the inhibition-zone diameter in the phase of RSM modeling of medium composition using *A. flavus* SA2BSS as test microorganism.

Response	SS	DF	MS	F-Value	*p*-Value	R^2^
Inhibition-zone diameter (mm)	23,139.19 ^a^ 5.25 ^b^	15.00 ^a^ 12.00 ^b^	1542.613 0.483 ^b^	3525.972	0.00	0.96

a—model; b—residual. SS—sum of squares; DF—degree of freedom; MS—mean squares; R^2^—coefficient of determination.

**Table 9:** ANOVA for the inhibition-zone diameter in the phase of RSM modeling of medium composition using *A. flavus* PA2DSS as test microorganism.

Response	SS	DF	MS	F-Value	*p*-Value	R^2^
Inhibition-zone diameter (mm)	20,035.22 ^a^ 4.56 ^b^	15.00 ^a^ 12.00 ^b^	1335.681 ^a^ 0.38 ^b^	3518.380	0.00	0.95

a—model; b—residual. SS—sum of squares; DF—degree of freedom; MS—mean squares; R^2^—coefficient of determination.

**Table 10:** The autoencoder network training results in ANN modeling.

Isolate	Metrics	Value
*Aspergillus flavus* SA2BSS	MSE	0.0016
	R^2^	0.969
*Aspergillus flavus* SA2BSS	MSE	0.0015
	R^2^	0.974

MSE—mean squared error; R^2^—coefficient of determination.

**Table 11:** Hyperparameters and results of model training using ANN methodology, with MSE and R^2^ calculated on the validation set.

Isolate	Neurons Number	Activation Function	Learning Rate	Learning Rate Degradation	MSE	R^2^
*Aspergillus flavus* SA2BSS	7	elu	0.020	0.85	0.00092	0.86
*Aspergillus flavus* PA2DSS	10	elu	0.035	0.70	0.00073	0.84

**Table 12:** RSM and ANN models comparison.

Model	Adjusted Coefficient of Determination (R^2^_adj_)
**RSM**	
*Aspergillus flavus* SA2BSS	0.91
*Aspergillus flavus* PA2DSS	0.89
**ANN**	
*Aspergillus flavus* SA2BSS	0.83
*Aspergillus flavus* PA2DSS	0.81

**Table 13:** Optimized values of varied factors and predicted values of inhibition-zone diameters.

**Factor**	**Goal**	**Optimized Value**
Cellulose	minimal	5.00
Urea	minimal	0.00
(NH_4_)_2_SO_4_ (g/L)	in range	3.77
K_2_HPO_4_ (g/L)	In range	0.50
**Response**	**Goal**	**Predicted value**
Inhibition-zone diameter (mm) *Aspergillus flavus* SA2BSS	maximal	3.66
Inhibition-zone diameter (mm) *Aspergillus flavus* PA2DSS	maximal	27.84

**Figure A1:**
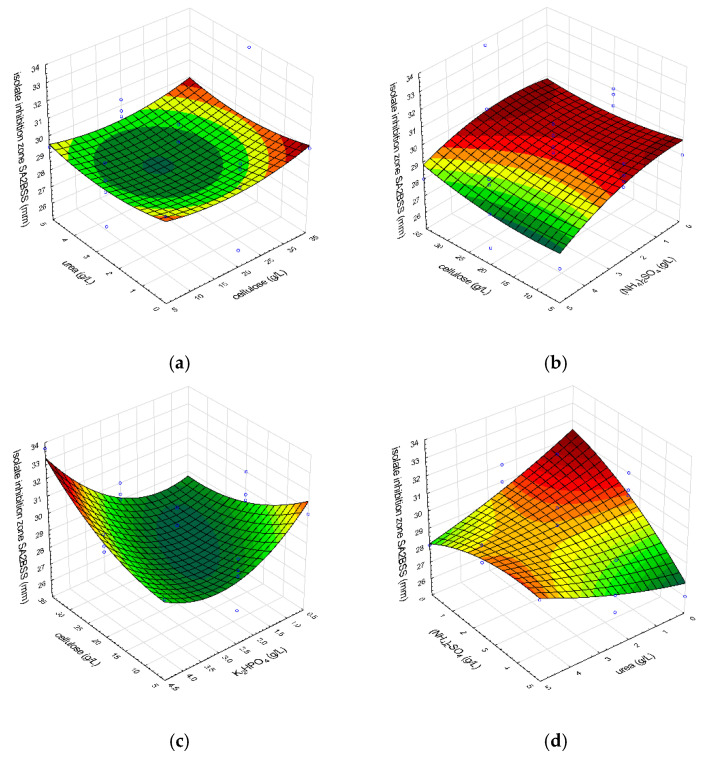

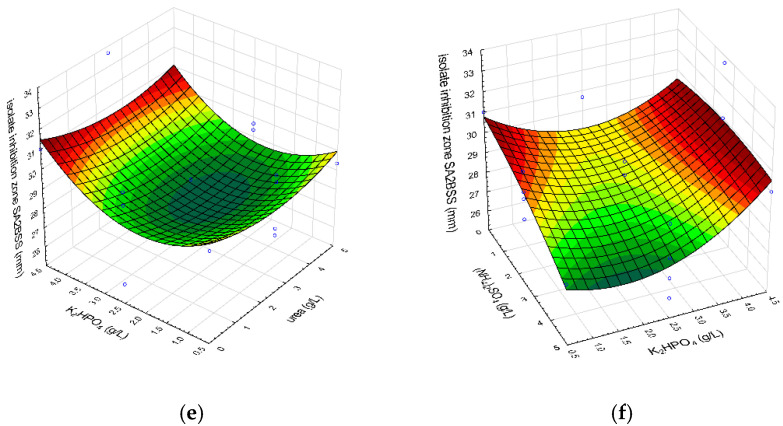
Response surface plots for effects of nutrients’ content on inhibition-zone diameter for *Aspergillus flavus* SA2BSS (RSM methodology): (**a**) cellulose and urea, (**b**) cellulose and (NH_4_)_2_SO_4_, (**c**) cellulose and K_2_HPO_4_, (**d**) urea and (NH_4_)_2_SO_4_, (**e**) K_2_HPO_4_ and urea, (**f**) K_2_HPO_4_, and (NH_4_)_2_SO_4_.

**Figure A2:**
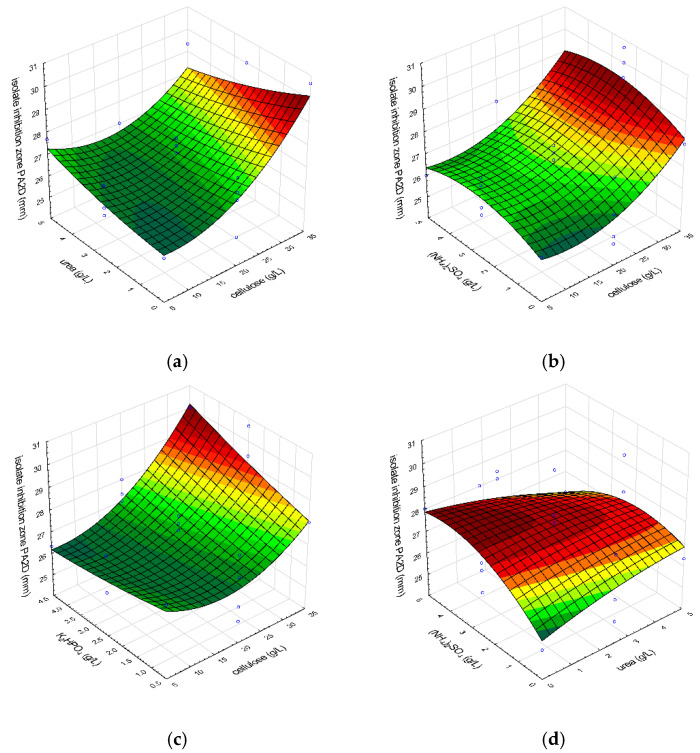

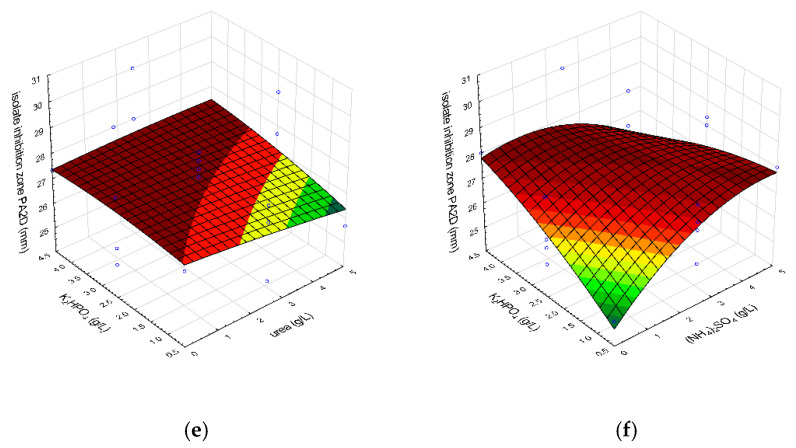
Response surface plots for effects of nutrients’ content on inhibition-zone diameter for *Aspergillus flavus* PA2DSS (RSM methodology): (**a**) cellulose and urea, (**b**) cellulose and (NH_4_)_2_SO_4_, (**c**) cellulose and K_2_HPO_4_, (**d**) urea and (NH_4_)_2_SO_4_, (**e**) K_2_HPO_4_ and urea, (**f**) K_2_HPO_4_, and (NH_4_)_2_SO_4_.

**Figure A3:**
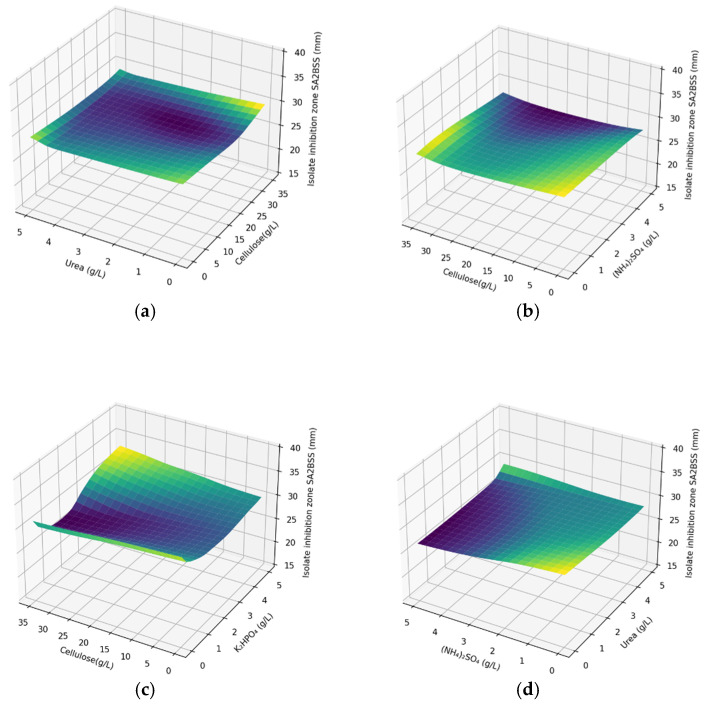

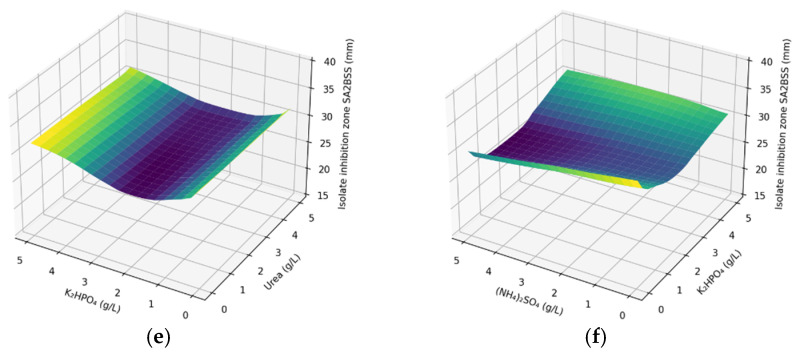
Response surface plots for effects of nutrients’ content on inhibition-zone diameter for *Aspergillus flavus* SA2BSS (ANN methodology): (**a**) cellulose and urea, (**b**) cellulose and (NH_4_)_2_SO_4_, (**c**) cellulose and K_2_HPO_4_, (**d**) urea and (NH_4_)_2_SO_4_, (**e**) K_2_HPO_4_ and urea, (**f**) K_2_HPO_4_, and (NH_4_)_2_SO_4_.

**Figure A4:**
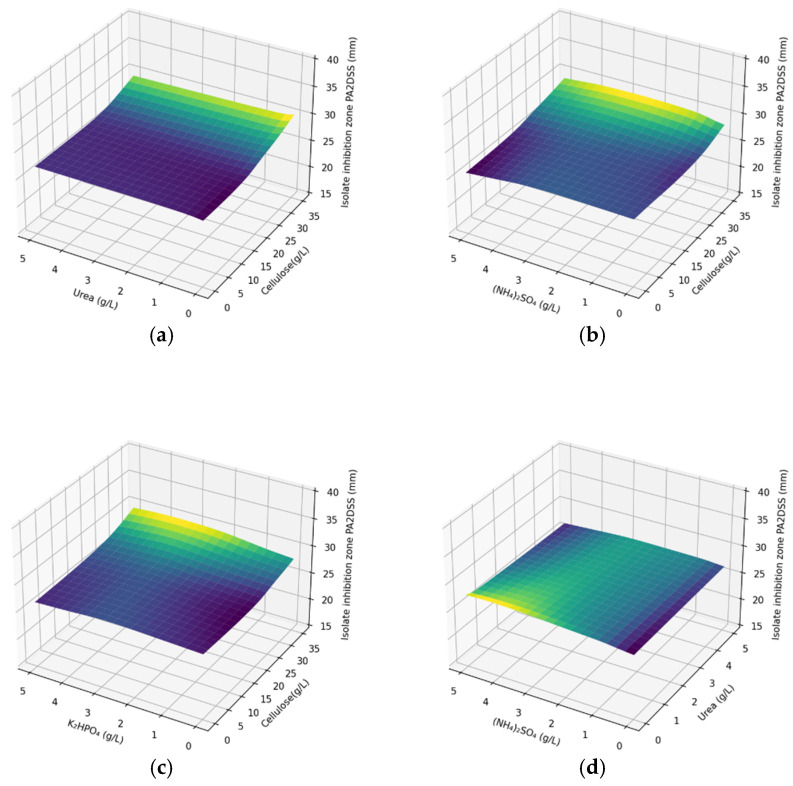

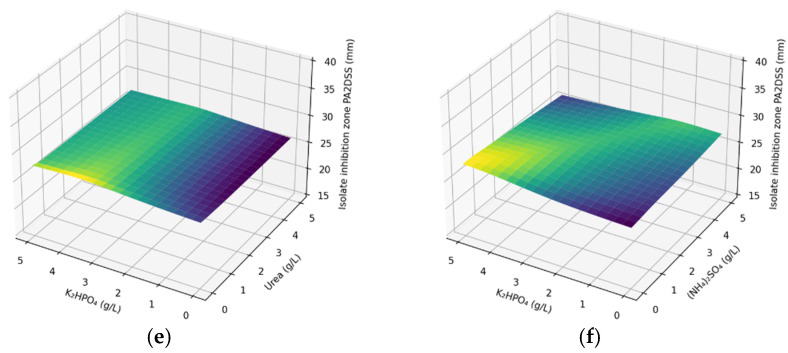
Response surface plots for effects of nutrients’ content on inhibition-zone diameter for *Aspergillus flavus* PA2DSS (ANN methodology): (**a**) cellulose and urea, (**b**) cellulose and (NH_4_)_2_SO_4_, (**c**) cellulose and K_2_HPO_4_, (**d**) urea and (NH_4_)_2_SO_4_, (**e**) K_2_HPO_4_ and urea, (**f**) K_2_HPO_4_, and (NH_4_)_2_SO_4_.

## Data Availability

Not applicable.

## Appendix A

**Table A1:** Box-Behnken experimental design = varied values of independent variables.

Cellulose Content (g/L)	Urea Content (g/L)	(NH_4_)_2_SO_4_ Content (g/L)	K_2_HPO_4_ Content (g/L)
5	0	2.5	2.5
35	0	2.5	2.5
5	5	2.5	2.5
35	5	2.5	2.5
20	2.5	0	0.5
20	2.5	5	0.5
20	2.5	0	4.5
20	2.5	5	4.5
5	2.5	2.5	0.5
35	2.5	2.5	0.5
5	2.5	2.5	4.5
35	2.5	2.5	4.5
20	0	0	2.5
20	5	0	2.5
20	0	5	2.5
20	5	5	2.5
5	2.5	0	2.5
35	2.5	0	2.5
5	2.5	5	2.5
35	2.5	5	2.5
20	0	2.5	0.5
20	5	2.5	0.5
20	0	2.5	4.5
20	5	2.5	4.5
20	2.5	2.5	2.5
20	2.5	2.5	2.5
20	2.5	2.5	2.5
